# Retracted: circCNN2 Accelerates Cell Proliferation and Invasion in Lung Squamous Cell Carcinoma via Regulating miR-184/E2F1 and Activating MAPK Signaling Pathway

**DOI:** 10.1155/2023/9757632

**Published:** 2023-08-02

**Authors:** Disease Markers

This article has been retracted by Hindawi following an investigation undertaken by the publisher [1]. This investigation has uncovered evidence of one or more of the following indicators of systematic manipulation of the publication process:

1. Discrepancies in scope
2. Discrepancies in the description of the research reported
3. Discrepancies between the availability of data and the research described
4. Inappropriate citations
5. Incoherent, meaningless and/or irrelevant content included in the article
6. Peer-review manipulation

The presence of these indicators undermines our confidence in the integrity of the article's content and we cannot, therefore, vouch for its reliability. Please note that this notice is intended solely to alert readers that the content of this article is unreliable. We have not investigated whether authors were aware of or involved in the systematic manipulation of the publication process.

Wiley and Hindawi regrets that the usual quality checks did not identify these issues before publication and have since put additional measures in place to safeguard research integrity.

We wish to credit our own Research Integrity and Research Publishing teams and anonymous and named external researchers and research integrity experts for contributing to this investigation.

The corresponding author, as the representative of all authors, has been given the opportunity to register their agreement or disagreement to this retraction. We have kept a record of any response received.

## References

[1] Hu S., Cao P., Kong K. (2022). circCNN2 Accelerates Cell Proliferation and Invasion in Lung Squamous Cell Carcinoma via Regulating miR-184/E2F1 and Activating MAPK Signaling Pathway. *Disease Markers*.

